# Development of a nomogram to predict recurrence scores obtained using Oncotype DX in Japanese patients with breast cancer

**DOI:** 10.1007/s12282-024-01616-z

**Published:** 2024-07-17

**Authors:** Akio Shibata, Nobuko Tamura, Keiichi Kinowaki, Aya Nishikawa, Kiyo Tanaka, Yoko Kobayashi, Takuya Ogura, Yuko Tanabe, Hidetaka Kawabata

**Affiliations:** 1 Department of Breast and Endocrine Surgery, Toranomon Hospital, 2-2-2 Toranomon, Minato City, Tokyo, 105-8470 Japan; 2 Department of Clinical Oncology, Toranomon Hospital, Tokyo, Japan; 3 Department of Pathology, Toranomon Hospital, Tokyo, Japan

**Keywords:** Breast cancer, Nomogram, Oncotype DX, Prediction, Recurrence

## Abstract

**Background:**

Chemotherapy is crucial for hormone receptor-positive, human epidermal growth factor receptor 2 (HER2)-negative breast cancer, and its survival benefits may outweigh adverse events. Oncotype DX (ODX) assesses this balance; however, it is expensive. Using nomograms to identify cases requiring ODX may be economically beneficial. We aimed to identify clinicopathological variables that correlated with the recurrence score (RS) and develop a nomogram that predicted the RS.

**Methods:**

We included 457 patients with estrogen receptor-positive, HER2-negative breast cancer with metastases in fewer than four axillary lymph nodes who underwent surgery and ODX at our hospital between 2007 and 2023. We developed nomograms and internally validated them in 310 patients who underwent surgery between 2007 and 2021 and validated the model’s performance in 147 patients who underwent surgery between 2022 and 2023.

**Results:**

Logistic regression analysis revealed that progesterone receptor (PgR) level, histological grade (HG), and Ki67 index independently predicted the RS. A nomogram was developed using these variables to predict the RS (area under the curve [AUC], 0.870; 95% confidence interval [CI], 0.82–0.92). The nomogram was applied to the model validation group (AUC, 0.877; 95% CI, 0.80–0.95). When the sensitivity of the nomogram was 90%, the model was able to identify 52.3% low-RS and 41.2% high-RS cases not requiring ODX.

**Conclusions:**

This was the first nomogram model developed based on data from a cohort of Japanese women. It may help determine the indications for ODX and the use of nomogram to identify cases requiring ODX may be economically beneficial.

**Supplementary Information:**

The online version contains supplementary material available at 10.1007/s12282-024-01616-z.

## Introduction

Breast cancer is one of the most common malignant diseases worldwide and was the most common cancer among women in 2020 [1]. Among Japanese women, breast cancer is the most frequently diagnosed cancer, with 97,142 cases and 14,650 deaths reported in 2019. The age-adjusted incidence rate of breast cancer in Japan has been increasing [2]. Adjuvant chemotherapy for hormone receptor (HR)-positive, human epidermal growth factor receptor 2 (HER2)-negative primary breast cancer, which accounts for approximately 70% of cases [3], is useful for reducing the risk of recurrence and improving survival prognosis. While chemotherapy is beneficial for reducing the risk of recurrence, its adverse effects, such as hair loss, fatigue, nausea, vomiting, immunosuppression, and drug-induced interstitial pneumonitis, can sometimes greatly outweigh the benefits of treatment. However, cases in which endocrine therapy alone is equally effective in reducing the risk of recurrence compared with adding chemotherapy to endocrine therapy have been reported; therefore, an accurate assessment of each patient’s risk of recurrence and requirement for chemotherapy is essential.

Oncotype DX (Genomic Health, Redwood City, CA; hereafter referred to as ODX) is a genetic test that calculates the recurrence score (RS) based on RNA expression levels of 21 genes (16 tumor-related genes and five reference genes) in tumor tissue from patients with invasive breast cancer [4–6]. This test predicts the prognosis and estimates the effects of chemotherapy. In the TAILORx study, chemotherapy could be safely omitted in the low-risk group (RS, 0–10) of patients without axillary lymph node metastases. The intermediate-risk group (RS, 11–25) revealed no effect of adding chemotherapy to endocrine therapy, while an RS within the range of 16–25 was associated with a prognostic benefit of chemotherapy only in patients under 50 years of age [7–9]. This indicated that in patients without axillary lymph node metastasis, a significant additive effect of chemotherapy existed if the RS was ≥ 26 and ≥ 16 in those with age > 50 years and ≦ 50 years, respectively. The RxPONDER study reported no additional benefit of chemotherapy in postmenopausal patients with an RS ≤ 25 in the group with metastases in 1–3 axillary lymph nodes. In premenopausal women, the addition of chemotherapy improved survival prognosis even if the RS was ≤ 25 [10].

Currently, ODX is recommended by several agencies, such as ASCO (American Society of Clinical Oncology) [11] and NCCN (National Comprehensive Cancer Network) [12], as a useful test. ODX is a valuable tool for determining the requirement of chemotherapy based on evidence. In the Japanese population, the RS has been shown to be useful for predicting prognosis based on distant recurrence-free interval, recurrence-free survival, and overall survival rates [13]. The breast cancer guidelines published by the Japanese Breast Cancer Society also strongly recommend omitting postoperative chemotherapy in patients with an RS ≤ 25 and no axillary lymph node metastasis. In Japan, the test is covered by insurance, with a copayment of US$900. Although ODX is expected to be used in clinical practice, the high cost of the test limits its availability. Identifying patients to be tested is important for reducing healthcare costs, as performing this test on patients at low clinical risk based on clinicopathological variables is not economical. Nomograms using clinicopathological variables have been reported to identify groups of patients who require chemotherapy [14–16]. However, these studies were conducted in Western populations and Asian women, excluding Japanese women, and data showing that these nomograms are predictive of recurrence in Japanese women are scarce. We performed a retrospective analysis of 457 patients who underwent ODX at our hospital to elucidate the clinicopathological variables useful for predicting high/low RS. Subsequently, a nomogram predicting the RS was developed based on the results obtained, and internal validation of the model’s performance was conducted. The nomogram was further adapted to another model validation group to verify the performance of the model.

## Materials and methods

### Patients and pathological evaluation of tumors

We retrospectively evaluated 457 patients with estrogen receptor (ER)-positive, HER2-negative, T1–3, N0–1 (metastases in less than four axillary lymph nodes), M0 (UICC TNM 8th Edition [17]) breast cancer who underwent surgery at our hospital between July 2007 and July 2023 and ODX. Consent for ODX was obtained from patients at the applicable stage. At the time of diagnosis, all patients were at least 20 years old. Patients who received neoadjuvant drug therapy were excluded.

Clinicopathological data were obtained from electronic medical records and included patient age, menopausal status, tumor size, number of axillary lymph nodes with metastases, stage, histological classification, histological grade (HG), nuclear grade (NG), lymphatic invasion, venous invasion, ER positivity, progesterone receptor (PgR) positivity, HER2 positivity, Ki67 expression, chemotherapy details, and the RS. Staging in all cases was performed according to the “TNM classification of Malignant Tumors” (8th Edition) [17]. HG was evaluated using the Elaston Ellis grading system [18], and ER positivity and PgR positivity were evaluated using the Allred scoring system based on the percentage of positive cells (proportion score) and staining intensity (intensity score) assessed using immunohistochemistry (IHC) [19, 20]. HER2 was considered negative in cases with scores 0 or 1 + on IHC and 2 + with no gene amplification on dual-color in situ hybridization (DISH) assessment (HER2/CEP17 < 2.0 and/or mean HER2 gene copy number per cell < 4.0) [21]. Ki67 was evaluated by hot spots in the staining area in immunostaining. Immunostaining for PgR, ER, HER2, and Ki67 was performed using Ventana BenchMark GX (Roche). The following antibodies were used: ER, clone SP1; PgR, clone 1E2; HER2, clone 4B5; and Ki67, clone 30–9. Ki67 index is an indicator of cell proliferative ability, and a value ≥ 20% indicates high proliferative ability [22].

### Statistical methods and model development and validation

The nomogram development group comprised 310 patients with breast cancer who underwent surgery between July 2007 and December 2021. The RS was classified as low (RS, 0–25) or high (RS, 26–100) according to the TAILORx study, and the chi-squared or Fisher’s exact test was used to compare clinicopathological variables between low and high RS groups. Univariate logistic regression analysis was used to analyze clinicopathological variables correlated with the RS, and multivariate logistic regression analysis was used to analyze variables with *p* < 0.05 in univariate analysis. Multicollinearity was confirmed for all variables. Nomograms were developed using variables that yielded significant results in the multivariate logistic regression analysis. Similarly, a nomogram predicting high RS was developed.

To verify the performance of the developed nomogram, a receiver operating characteristic (ROC) curve was plotted, and the area under the curve (AUC) was calculated. Further calibration was performed to assess discrepancies between the actual RS and the nomogram predictions. The performance of the model was evaluated by adapting the developed nomograms to the group of 147 patients with breast cancer who underwent surgery between January 2022 and July 2023 (the model validation group).

Assuming that the developed nomograms would be used for screening the eligibility for implementing ODX, a prediction probability of 90% sensitivity was calculated from the ROC curve. Based on this prediction probability, we also evaluated the predictive performance in terms of sensitivity and specificity to identify patients in whom ODX can be omitted. All statistical tests were two-sided, and statistical significance was set at *p* < 0.05. All statistical analyses were performed using R version 4.0.2 (R Core Team (2020); R: Language and environment for statistical computing. R Foundation for Statistical Computing, Vienna, Austria; URL: https://www.R-project.org/). This study was conducted according to the “TRIPOD (Transparent Reporting of a Multivariable Prediction Model for Individual Prognosis or Diagnosis) statement” [23].

### Ethical statement

The relevant ethics committee approved the study protocol (registration number: 1552). The requirement for informed consent was waived owing to the retrospective and anonymous nature of this study.

## Results

### Patient characteristics

The mean age of the patients was 51.4 years, which was significantly higher in the high RS group than in the low RS group (*p* = 0.002). The high RS group had a significantly higher number of postmenopausal patients (*p* < 0.001), lower PgR level (*p* < 0.001), higher HG (*p* < 0.001), and higher Ki67 index (*p* < 0.001) than the low RS group. No statistically significant difference in lymph node metastasis status existed between the two RS groups (*p* = 0.231). Regarding the histological type, 279 cases (90.0%) were invasive ductal carcinomas, 19 cases (6.1%) were invasive lobular carcinomas, and 12 cases (3.9%) exhibited other features. Two hundred and sixty-five (85.5%) and 45 (14.5%) patients had low and high RS values, respectively. The patient characteristics of the nomogram development group are shown in Table 1.

**Table 1:** Characteristics of the model development group and validation group

	Model development group (*n* = 310)			Validation group (*n* = 147)		
	RS 0–25 (*n* = 265)	RS 26–100 (*n* = 45)	*p* value	RS 0–25 (*n* = 130)	RS 26–100 (*n* = 17)	*p* value
Age(mean)	50.77	55.38	0.002	51.11	56.00	0.073
Menopause (%)			< 0.001			0.179
Pre	172 (64.9)	12 (26.7)		80 (61.5)	7 (41.2)	
Post	93 (35.1)	33 (73.3)		50 (38.5)	10 (58.8)	
Invasive size (%)			0.177			0.041
≦20 mm	210 (79.2)	31 (68.9)		108 (83.1)	10 (58.8)	
> 20 mm	55 (20.8)	14 (31.1)		22 (16.9)	7 (41.2)	
LN metastasis (%)			0.231			0.022
Negative	179 (67.5)	35 (77.8)		92 (70.8)	17 (100)	
Positive	86 (32.5)	10 (22.2)		38 (29.2)	0 ( 0.0)	
Stage (%)			0.712			0.331
IA	147 (55.5)	24 (53.3)		77 (59.2)	10 (58.8)	
IB	12 ( 4.5)	1 ( 2.2)		9 ( 6.9)	0 ( 0.0)	
IIA	78 (29.4)	13 (28.9)		35 (26.9)	7 (41.2)	
IIB	28 (10.6)	7 (15.6)		9 ( 6.9)	0 ( 0.0)	
Histological class (%)			0.491			0.364
IDC	237 (89.4)	42 (93.3)		116 (89.2)	17 (100.0)	
ILC	18 ( 6.8)	1 ( 2.2)		12 ( 9.2)	0 ( 0.0)	
Others	10 ( 3.8)	2 ( 4.4)		2 ( 1.5)	0 ( 0.0)	
HG (%)			< 0.001			0.014
1	82 (30.9)	3 ( 6.7)		30 (23.1)	2 (11.8)	
2	177 (66.8)	36 (80.0)		100 (76.9)	14 (82.4)	
3	6 ( 2.3)	6 (13.3)		0 ( 0.0)	1 ( 5.9)	
Ly (%)			0.333			0.015
Negative	166 (62.6)	27 (60.0)		80 (61.5)	8 (47.1)	
Positive	89 (33.6)	18 (40.0)		50 (38.5)	8 (47.1)	
NA	10 ( 3.8)	0 ( 0.0)		0 ( 0.0)	1 ( 5.9)	
V (%)			0.110			0.020
Negative	241 (90.9)	45 (100.0)		129 (99.2)	16 (94.1)	
Positive	14 ( 5.3)	0 ( 0.0)		1 ( 0.8)	0 ( 0.0)	
NA	10 ( 3.8)	0 ( 0.0)		0 ( 0.0)	1 ( 5.9)	
ER score (%)			0.062			0.001
5	0 ( 0.0)	1 ( 2.2)		0 ( 0.0)	1 ( 5.9)	
6	8 ( 3.0)	2 ( 4.4)		0 ( 0.0)	1 ( 5.9)	
7	33 (12.5)	8 ( 17.8)		2 ( 1.5)	0 ( 0.0)	
8	224 (84.5)	34 ( 75.6)		128 ( 98.5)	15 ( 88.2)	
PgR score (%)			< 0.001			< 0.001
0	11 ( 4.2)	11 (24.4)		1 ( 0.8)	2 (11.8)	
2	4 ( 1.5)	2 ( 4.4)		2 ( 1.5)	2 (11.8)	
3	8 ( 3.0)	5 (11.1)		1 ( 0.8)	0 ( 0.0)	
4	16 ( 6.0)	3 ( 6.7)		0 ( 0.0)	1 ( 5.9)	
5	20 ( 7.5)	6 (13.3)		2 ( 1.5)	1 ( 5.9)	
6	31 (11.7)	8 (17.8)		10 ( 7.7)	3 (17.6)	
7	49 (18.5)	3 ( 6.7)		19 (14.6)	1 ( 5.9)	
8	126 (47.5)	7 (15.6)		95 (73.1)	7 (41.2)	
Ki67 (%)			< 0.001			< 0.001
< 20%	181 (68.3)	10 (22.2)		66 (50.8)	0 (0)	
≧20%	84 (31.7)	35 (77.8)		64 (49.2)	17 (100.0)	
Chemo therapy (%)			< 0.001			< 0.001
None	248 (93.6)	11 (24.4)		121 (93.1)	0 ( 0.0)	
Do	16 ( 6.0)	34 (75.6)		8 ( 6.2)	17 (100.0)	
NA	1 ( 0.4)	0 ( 0.0)		1 ( 0.8)	0 ( 0.0)	

Abbreviations: *RS* recurrence score, *LN* lymph node, *IDC* invasive ductal carcinoma, *ILC* invasive lobular carcinoma, *HG* histological grade, *Ly* lymphatic invasion, *V* venous invasion, *ER* estrogen receptor, *PgR* progesterone receptor, *NA* not applicable

### Development of nomograms

**Fig. 1:**
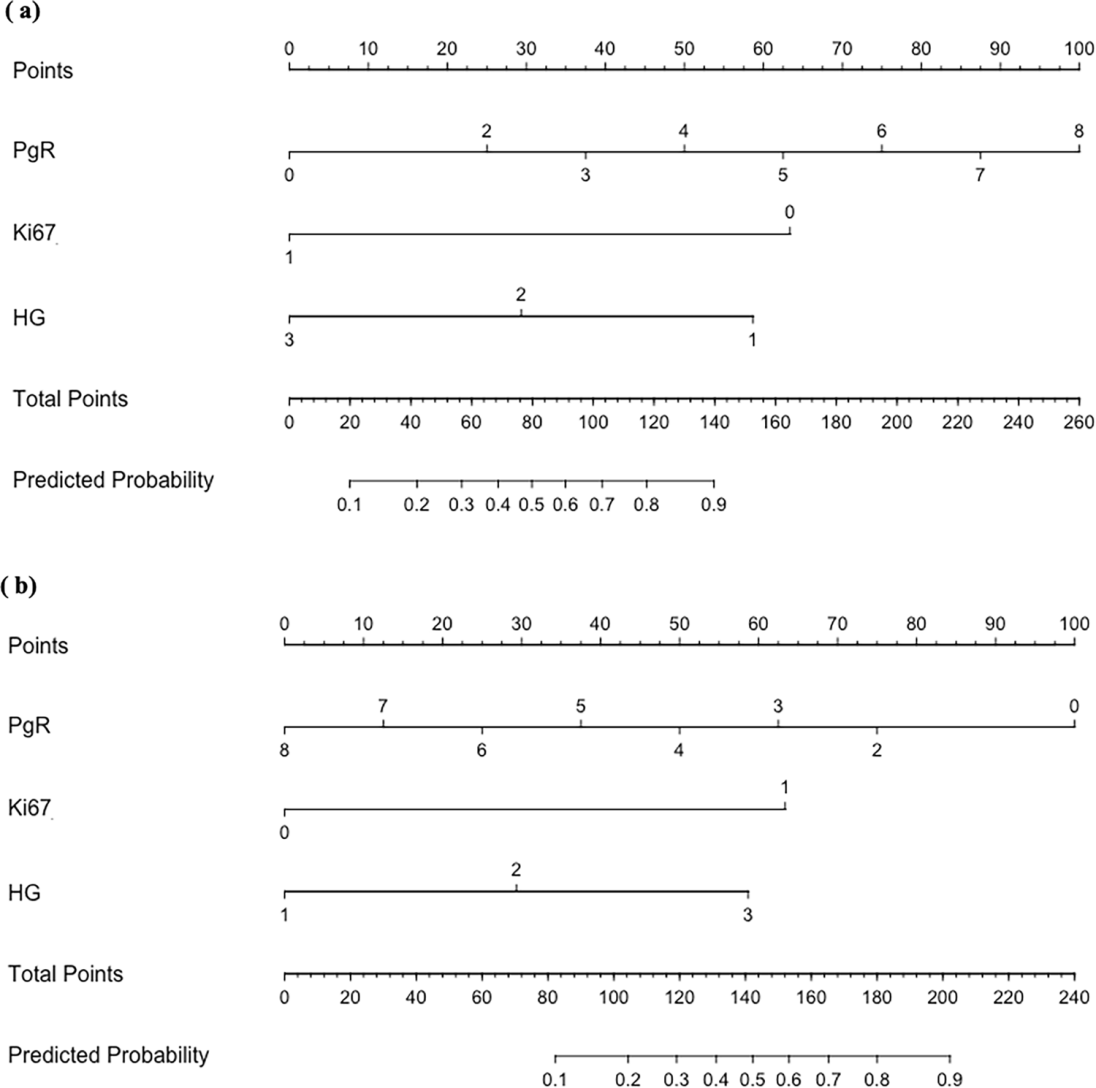
Nomogram to predict the probability of low recurrence score (**a**) and high recurrence score (**b**). The nomogram was developed using variables that were significant in the multivariate logistic regression analysis

Univariate logistic regression analysis revealed that premenopausal status, a high PgR level, a low HG, and a low Ki67 index were significantly associated with a low RS. Ki67 index showed the strongest statistical significance with a low RS (odds ratio = 0.113). Multivariate regression analysis of variables that were significantly different from univariate regression analysis showed that a high PgR level, low HG, and low Ki67 index were independent predictors of a low RS. The odds ratio, 95% confidence interval (CI), and *p*-value for the RS and each clinicopathological variable are shown in Table 2. A nomogram was developed to predict low RS values using variables that yielded significant results in multivariate logistic regression analysis (Fig. 1a). Similarly, we developed a nomogram to predict high RS values (Fig. 1b).

**Table 2:** Univariate and multivariate logistic regression analysis for each clinicopathological factor to predict recurrence score in the model development group

		Univariate analysis		Multivariate analysis		
	Odds ratio	95% CI	*p* value	Odds ratio	95% CI	*p* value
Menopause	0.205	0.097–0.407	< 0.001	0.506	0.217–1.180	0.114
PgR	1.448	1.281–1.646	< 0.001	1.500	1.270–1.780	< 0.001
Ki67	0.138	0.062–0.282	< 0.001	0.113	0.044–0.291	< 0.001
HG	0.177	0.072–0.384	< 0.001	0.341	0.123–0.947	0.039
Ly	0.804	0.423–1.560	0.511			
V	7.944^e+06^	1.456^e−24^-NA	0.988			
Invasive size	0.986	0.962–1.013	0.270			
Node positive	1.750	0.855–3.884	0.143			

PgR, HG, and Ki67 were independent predictors of RS

Abbreviations: *CI* confidence interval, *PgR* progesterone receptor, *HG* histological grade, *Ly* lymphatic invasion, *V* venous invasion

### Evaluation of model performance

**Fig. 2:**
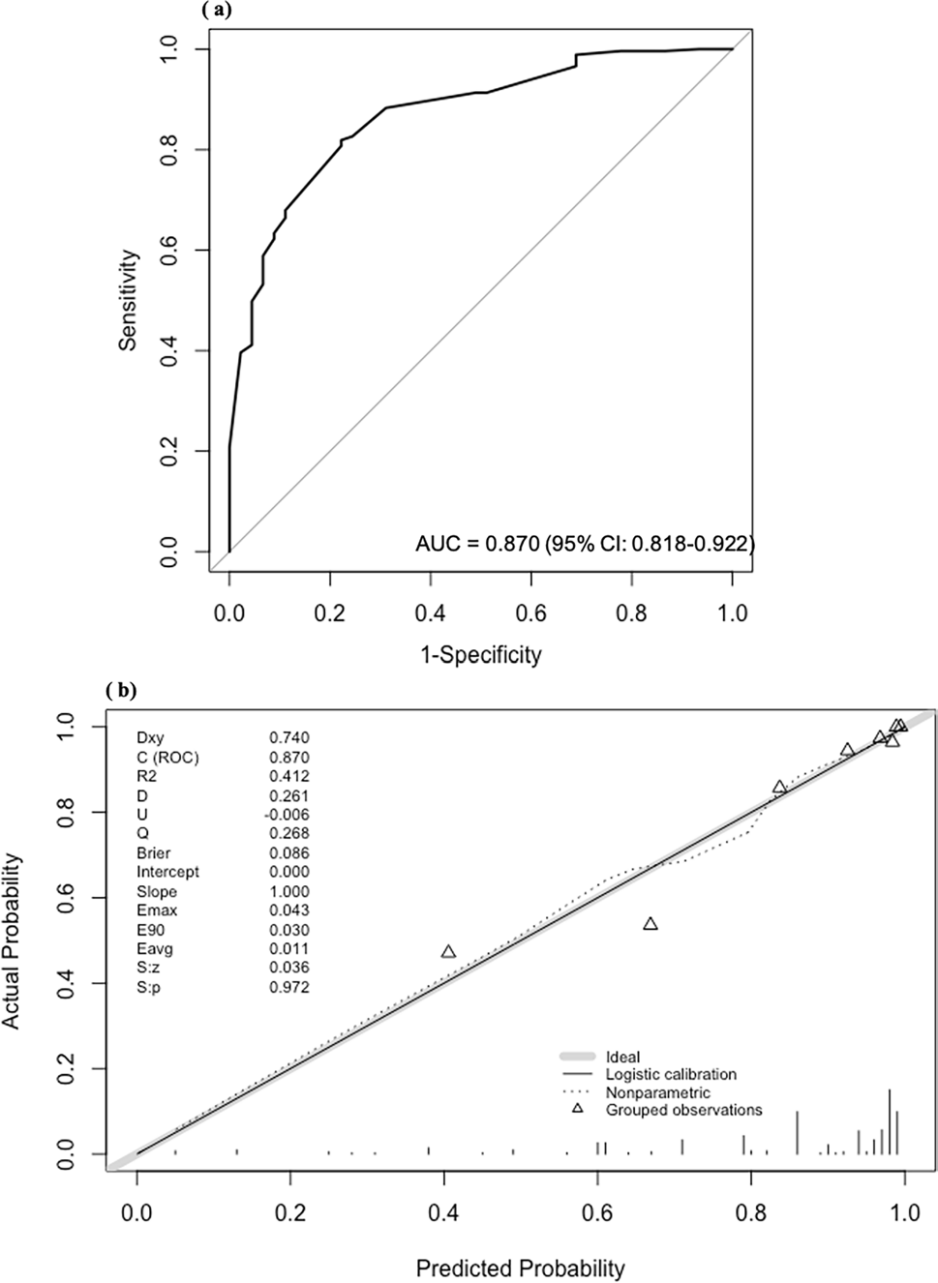
Receiver operating characteristic curve of nomogram (**a**) and calibration plot (**b**) in the model development group

An ROC curve was plotted to confirm the discriminatory ability of the nomogram (AUC [C-index] = 0.870 [95% CI, 0.82–0.92]; Fig. 2a). Further calibration was performed to check for discrepancies between the actual RS and the predicted value of the nomogram (Fig. 2b).

**Fig. 3:**
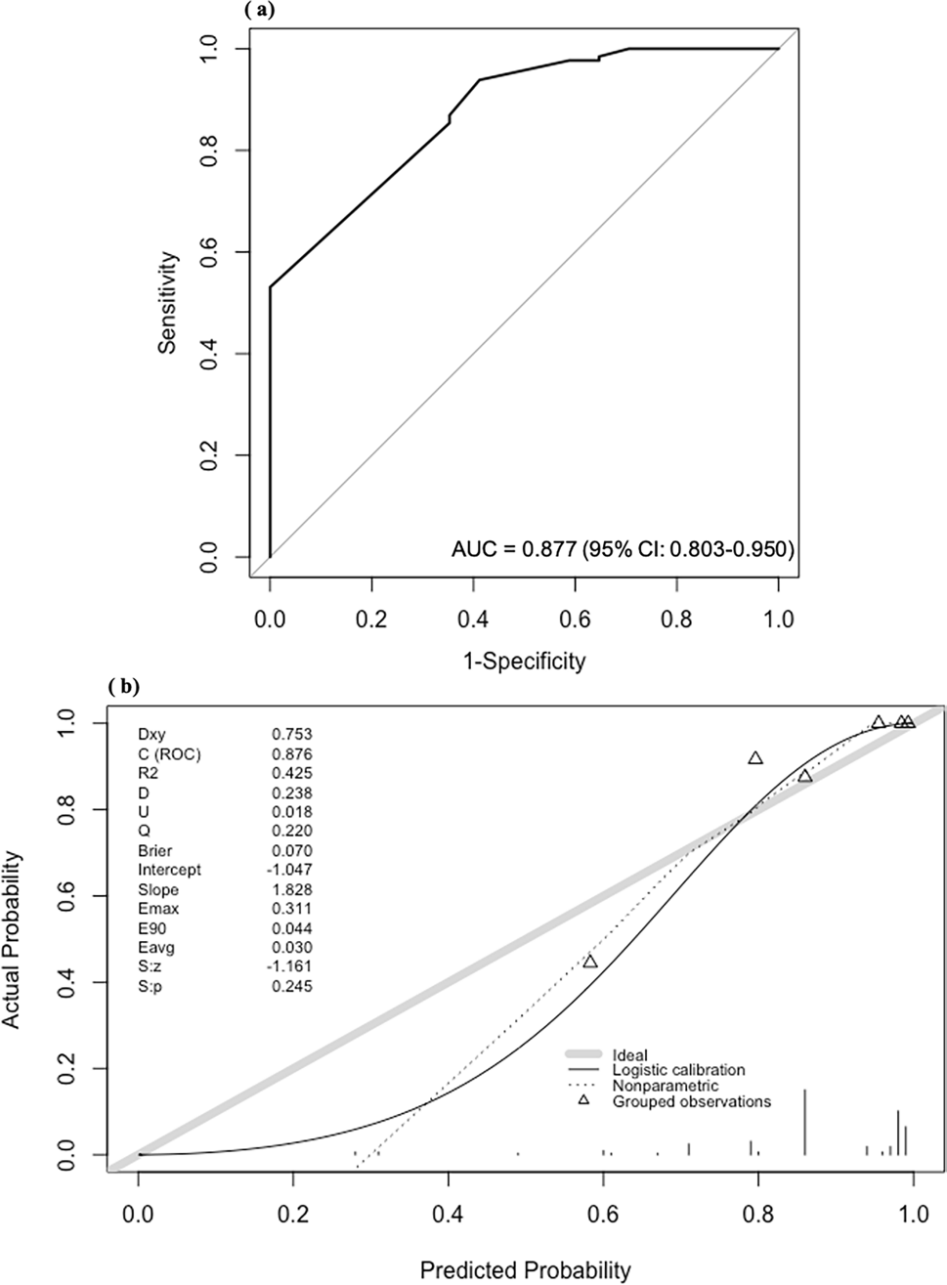
Receiver operating characteristic curve (**a**) and calibration plot (**b**) in the validation group. The discriminative ability was equivalent to that of the model development group

The nomogram was then adapted to the model validation group to verify the prediction accuracy of the model. Similar to the nomogram development group, statistically significant differences in low PgR levels (*p* < 0.001), high HG (*p* = 0.014), and high Ki67 values (*p* < 0.001) were observed. In addition, statistically significant differences in lymph node metastasis status, ER level, venous invasion, and lymphatic invasion were present. Patient characteristics of the model validation group are shown in Table 1. The regression coefficients obtained using multivariate logistic regression analysis of the data of the nomogram development group were adapted to the model validation group to calculate the predicted probability of a low RS. An ROC curve was plotted to confirm the discriminative ability of the low RS in the model validation group based on the obtained predictive probabilities, and the result (AUC = 0.877 [95% CI, 0.80–0.95]) was equivalent to the discriminative ability of the nomogram development group (Fig. 3a). The calibration results of the model validation group are shown in Fig. 3b.

The ROC curve of the nomogram development group showed that the nomogram predicting a high RS had a predictive probability of 8.6% at a sensitivity of 90% for discriminating RS. Similarly, the nomogram predicting a low RS had a predictive probability of 69.4% at a sensitivity of 90%. The sensitivity and specificity of the high and low RS values, calculated using these cutoff values, of the model validation group are shown in Table 3. Using a nomogram that predicted a high RS, we estimated that 52.3% of cases did not require chemotherapy. Similarly, using a nomogram that predicted a low RS, we estimated that 41.2% of cases required chemotherapy.

**Table 3:** Sensitivity and specificity of the model validation group calculated from the predicted probability of a low or high RS nomogram with a sensitivity of 90%

ODX RS			
		0–25 (*n* = 130)	26–100 (*n* = 17)
Predicted probability of low RS (cutoff value is 69.4%)	High	127	10
	Low	3	7
Sensitivity		97.7%	58.8%
Specificity		2.3%	41.2%

ODX RS			
		26–100 (*n* = 17)	0–25 (*n* = 130)
Predicted probability of high RS (cutoff value is 8.6%)	High	17	62
	Low	0	68
Sensitivity		100%	47.7%
Specificity		0%	52.3%

In the model validation group, low or high RS nomograms were able to estimate the need for ODX in 41.2% and 52.3% of cases, respectively

*ODX* oncotype DX, *RS* recurrence score

## Discussion

The results of the present study are valuable because the study represents a single-center analysis of ODX in a large number of Japanese patients with breast cancer. The study used pathological parameters commonly assessed in clinical practice, which enhanced the convenience of the model. To the best of our knowledge, no nomograms have been created based on data from Japanese patients with breast cancer. In the future, such nomograms will help identify groups of patients who require chemotherapy at a low cost and select appropriate treatment for patients who cannot undergo ODX for financial reasons.

The present study revealed that PgR level, Ki67 index, and HG were significant predictors of the RS, consistent with previous studies [14–16, 24, 25]. In particular, PgR and Ki67 genes are the target genes of ODX, and their correlation has been advocated. A single-center study reported a correlation between PgR positivity and the RS. The study reported that patients who were PgR-negative had a significantly higher RS than those who were PgR-positive and a statistically significant correlation between the RS and PgR positivity and HG [26]. Allison et al. reported that PgR positivity, Ki67 index, and NG correlated with the RS. They observed that ODX had little benefit in cases of low clinical risk (grade 1, high PgR level (AS ≥ 5), Ki67 ≤ 10%) or high clinical risk (grade 3, low PgR level (< 5), Ki67 > 10%) [27]. The study conducted by Onoda et al. in the Japanese population also suggested a similar correlation between the RS and PgR level, Ki67 index, and NG. An analysis of 139 cases from a single center revealed a negative correlation between the RS and PgR level (r =   −  0.53), while NG (r = 0.41) and Ki67 index (r = 0.42) were positively correlated with the RS [28]. These results suggest that PgR level, Ki67 index, and grade are useful predictors of the RS in different ethnic groups.

Several alternative predictive tools to ODX have been studied in different countries, and various nomograms have been developed. Three types of Magee equation (ME) (ME1–ME3) are used to predict the RS obtained using ODX. Each equation uses different variables. When the total scores calculated using these equations were < 18 or > 31, the model showed 98% classification performance in predicting the RS (≤ 25 or > 25) [14]. The nomogram developed at Tennessee University is a model based on the NCDB database that was able to discriminate the RS (≤ 25 or > 25) with a high accuracy (C-index = 0.81). When the predictive probability of a high/low RS was between 85 and 100%, the model could predict a high or low RS in 92.7% of cases [15]. However, according to a Korean report, the nomogram developed at Tennessee University cannot accurately predict RS in Asian populations, and developing a nomogram using datasets from various races is essential [29]. However, reportedly, nomograms have been created based on data from Asian populations. Yoo et al. developed a nomogram predicting an RS > 25, and NG, PgR level, and Ki67 index were incorporated into the model since these correlated with the RS. The model showed good discriminative ability with an AUC of 0.828 in the validation cohort [24]. Similarly, Kim et al. also developed a model with good discriminatory ability (AUC = 0.926) using NG, Ki67 index, and PgR level [25]. Lee et al. developed a nomogram based on data from 485 patients with breast cancer metastases in 0–3 axillary lymph nodes. The model included NG, lymphatic invasion, ER level, PgR level, and Ki67 index as variables that correlated with a low RS (RS < 25) and showed high discriminatory power (AUC = 0.88) [19]. Davey et al. developed a similar nomogram using data from 448 patients with breast cancer with negative axillary lymph nodes. This nomogram included factors such as menopausal status, stage, and symptoms, in addition to ER level, PgR level, and grade [30]. Although these nomograms have some differences in variables, as discussed above, PgR level, grade, and Ki67 index are highly correlated with the RS. These variables were included in the model. The nomogram developed at our hospital had results similar to those of previously reported nomograms. Most of the previously reported nomograms are intended to predict a high RS (RS ≧ 26) and low RS (RS ≦ 25); however, the TAILORx trial reported a survival benefit of chemotherapy in patients younger than 50 years and with an RS within 16–25. Further research is required to determine the RS cutoff value that the nomogram predicts. We examined whether a model could be developed to predict RS 16 in our cohort of patients under 50 years old, who were suggested to potentially benefit from the TAILORx trial of add-on chemotherapy. A similar analysis was conducted on 115 patients under 50 years old, who were negative for lymph node involvement, but no model could be developed to predict RS 16 (AUC = 0.641 [95% CI, 0.489–0.794]) (Supplements Table 1, Fig. 2a, Fig. 2b). We also explored the potential of development models for premenopausal patients and cases under 50 years old, regardless of lymph node status, but were unable to produce nomograms. If the cutoff value of RS is set at 16, it is presumed that the differences in tumor biology, especially proliferative potential, above and below that value are small, making it difficult to develop a predictive model of RS using clinicopathological factors. Also, the combination of PgR, HG, and Ki67 was still unable to predict RS values below 15 and above 16. Therefore, our nomogram is a valid model when the RS cutoff is 26 and cannot be used to predict low RS values in node-negative patients under 50 years of age, and it is important to understand this limitation when using this model.

The cost-effectiveness of ODX has been verified in several countries. Yamauchi et al. reported that ODX was socially cost-effective based on quality-adjusted life-years (QALYs) of a Japanese cohort [31]. This study reported that ODX reduced the frequency of chemotherapy by 19%, extended the QALY per patient by 0.241 years, and had a cost-effectiveness ratio of US$6,368 per QALY. In particular, in the high-risk groups, chemotherapy reduced the cost of treating recurrent disease and extended survival so that the survival benefit outweighed the cost of ODX. Hornberger et al. evaluated the economic impact of ODX in the US [32]. This study examined the impact of ODX on the decision to administer chemotherapy and evaluated the cost-effectiveness of the test. In a cohort of 100 patients, ODX extended the QALY by 8.6 years and reduced the total cost by US$202,828. Wang et al. reviewed the cost-effectiveness of ODX and reported that when evaluating the cost-effectiveness of ODX, it is important to consider commonly used clinicopathological variables [33]. PREDICT (http://www.predict.nhs.uk) [34, 35] was used to classify clinical risk based on the estimated reduction in mortality with chemotherapy and to compare the QALYs in each risk group. Compared to patients at low clinical risk, the incremental cost-effectiveness ratio of ODX has been shown to be high in intermediate- and high-risk patients ($124,600 [low], $28,700 [medium], and $15,700 [high] per QALY) [36]. Since these studies suggest that ODX is particularly cost-effective in cases of high clinical risk, using nomograms that consider clinicopathologic factors in cases in which ODX is indicated may be useful from a health-economic perspective. Furthermore, in recent years, expensive drugs, such as CDK4/6 inhibitors, have been increasingly used for treating the recurrent disease. Future cost-effectiveness studies should consider the potential underestimation of post-recurrence treatment costs related to these new drugs.

The present study has several limitations. First, this was a single-center cohort study, which might have introduced bias in patient selection. The sample population analyzed may not be representative of all patients with HR + /HER2 − breast cancer as ODX is not always performed in cases where clinicopathological variables can predict a high or low risk of recurrence, older adult patients, patients with poor performance status, or patients who decline testing owing to financial or personal reasons. However, these reasons are also common in other centers and can be generalized with respect to cohort selection. Second, in the present study, the cutoff value was set at 90% to use the developed nomogram for screening for eligibility for undergoing ODX. Verifying the optimal cutoff value prospectively in a population that has been screened for eligibility for undergoing ODX using the developed nomogram is essential. Although uniform determination of cutoff value was not possible, we judged it to be a reasonable value that would not excessively decrease the specificity while preventing the omission of cases during screening. The developed nomogram may be increasingly applicable to the Japanese population if it is further validated at other centers.

In conclusion, we developed a nomogram based on the PgR level, HG, and Ki67 index to predict the RS obtained using ODX. This is the first model based on data from Japanese patients with breast cancer, which has allowed us to determine the indications for ODX in approximately half of the patients with ER-positive, HER2-negative disease with metastases in less than four axillary lymph nodes. These findings seem useful in terms of healthcare economics.

## Supplementary Information

Below is the link to the electronic supplementary material.

Supplementary file1 (DOCX 14 KB)

Supplementary file2 (TIFF 1909 KB) Supplement 2 Receiver operating characteristic curve of nomogram (supplement 2a) and calibration plot (supplement 2b) in the model development group. The predictive ability of the model with RS 16 as the cutoff was poor. Abbreviations: AUC; area under the curve, CI; confidence interval

Supplementary file3 (TIFF 2694 KB) Supplement 2 Receiver operating characteristic curve of nomogram (supplement 2a) and calibration plot (supplement 2b) in the model development group. The predictive ability of the model with RS 16 as the cutoff was poor. Abbreviations: AUC; area under the curve, CI; confidence interval

## Data Availability

The dataset generated and analyzed during the current study are available from the corresponding author on reasonable request.
