# Supplementary material for: Development of a nomogram to predict recurrence scores obtained using Oncotype DX in Japanese patients with breast cancer
Source: Breast Cancer. 2024 Jul 17;31(6):1018–27. doi: 10.1007/s12282-024-01616-z (PMC11489311; doi:10.1007/s12282-024-01616-z)
Supplement: Supplementary file 1 — Supplementary file1 (DOCX 14 KB) [file 12282_2024_1616_MOESM1_ESM.docx]

**Supplement 1** Univariate and multivariate logistic regression analysis for each clinicopathological factor to predict recurrence score in the model development group. RS 16 was set as the cutoff for this analysis. HG, and Invasive size were independent predictors of RS 16

|  |  | Univariate analysis |  | Multivariate analysis | | |
| --- | --- | --- | --- | --- | --- | --- |
|  | Odds ratio | 95% CI | *p* value | Odds ratio | 95% CI | *p* value |
| Menopause | 6.564e+06 | 4.726e-64-NA | 0.988 |  |  |  |
| PgR | 0.900 | 0.711-1.123 | 0.356 |  |  |  |
| Ki67 | 1.714 | 0.793-3.756 | 0.173 |  |  |  |
| HG | 3.490 | 1.548-8.575 | 0.004 | 3.063 | 1.257-8.123 | 0.018 |
| Ly | 3.333 | 1.381-8.572 | 0.009 | 2.480 | 0.966-6.676 | 0.063 |
| V | 0.637 | 0.125-2.734 | 0.551 |  |  |  |
| Invasive size | 1.055 | 1.013-1.110 | 0.021 | 1.043 | 1.004-1.093 | 0.041 |

Abbreviations : CI; confidence interval, PgR; progesterone receptor, HG; histological grade, Ly; lymphatic invasion, V; venous invasion
