# Supplementary figures and images for: Development of a nomogram to predict recurrence scores obtained using Oncotype DX in Japanese patients with breast cancer
Source: Breast Cancer. 2024 Jul 17;31(6):1018–27. doi: 10.1007/s12282-024-01616-z (PMC11489311; doi:10.1007/s12282-024-01616-z)

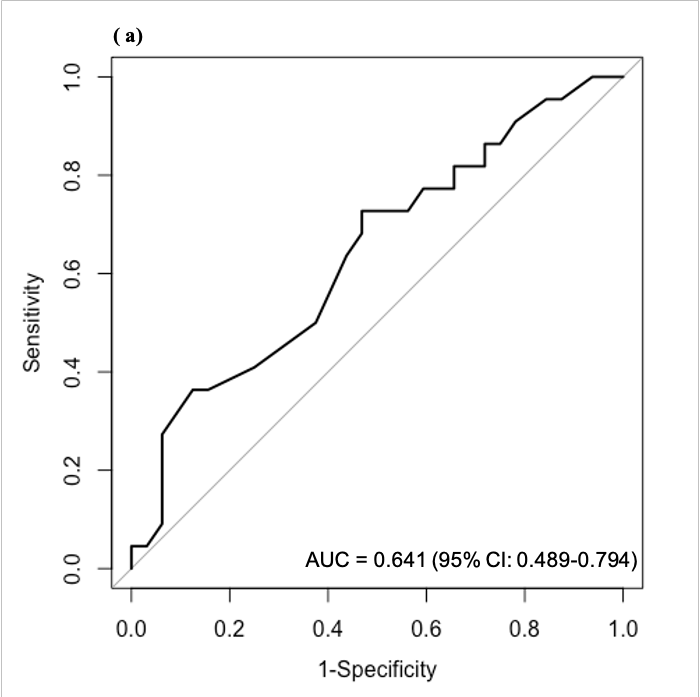

Supplement: Supplementary file 2 — Supplementary file2 (TIFF 1909 KB) Supplement 2 Receiver operating characteristic curve of nomogram (supplement 2a) and calibration plot (supplement 2b) in the model development group. The predictive ability of the model with RS 16 as the cutoff was poor. Abbreviations: AUC; area under the curve, CI; confidence interval [file 12282_2024_1616_MOESM2_ESM.tiff]

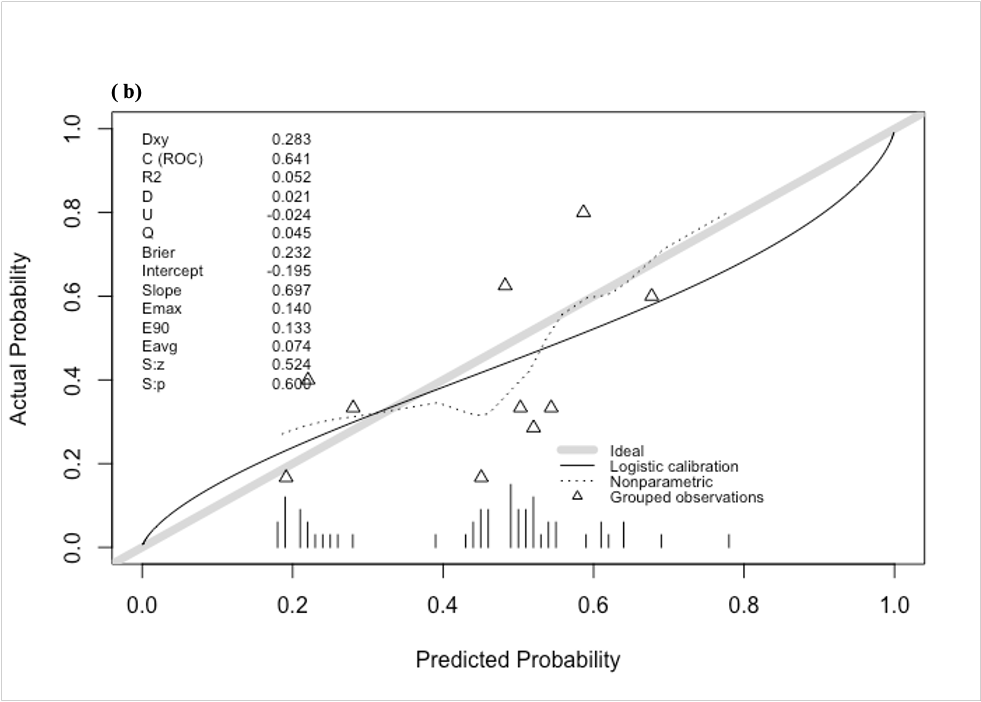

Supplement: Supplementary file 3 — Supplementary file3 (TIFF 2694 KB) Supplement 2 Receiver operating characteristic curve of nomogram (supplement 2a) and calibration plot (supplement 2b) in the model development group. The predictive ability of the model with RS 16 as the cutoff was poor. Abbreviations: AUC; area under the curve, CI; confidence interval [file 12282_2024_1616_MOESM3_ESM.tiff]
